# Light- and drug-induced pupillary dynamics in eyes with a retropupillary iris-claw intraocular lens

**DOI:** 10.1007/s00417-023-06025-0

**Published:** 2023-03-02

**Authors:** Carlo Bellucci, Andrea Perrella, Maurizio Rossi, Annalaura Papapicco, Federico Spadini, Salvatore Antonio Tedesco, Stefano Gandolfi, Paolo Mora

**Affiliations:** 1 Ophthalmology Unit, Department of Medicine and Surgery, University Hospital of Parma, Via Gramsci 14, 43126 Parma, Italy; 2 Department of Clinical and Experimental Medicine, University Hospital of Parma, Parma, Italy

**Keywords:** Cataract surgery, Iris-claw IOL, Pupillary dynamics, Pharmacological mydriasis, Pharmacological miosis

## Abstract

**Purpose:**

We evaluated the pupillary characteristics and response to light and drugs in eyes with posterior chamber (PC) placement of iris-claw intraocular lens (IC-IOL).

**Methods:**

In this cross-sectional, comparative study, we included adults with an IC-IOL implanted in the PC of a single eye. We excluded patients with ocular trauma, postoperative IC-IOL displacement or complications, and extended iris atrophy. We used anterior segment optical coherence tomography to perform light-controlled pupillography, measure the pupil diameter (PD), and estimated the pupil circularity under mesopic conditions. PD was also assessed under photopic, scotopic, pharmacological mydriasis, and miosis conditions. The results were compared to those of the fellow eye, phakic, or regular pseudophakic.

**Results:**

The IC-IOL and control groups included 30 eyes each. The most frequent reasons for IC-IOL implantation were complicated cataract (37%) and dislocated/luxated prior IOL (33%). Compared to the control group, the IC-IOL group had lower visual acuity, a smaller PD under scotopic conditions (*p* = 0.0010) and after pharmacological mydriasis (*p* < 0.0001), and a larger PD after pharmacological miosis (*p* < 0.0001). Mesopic pupil circularity was comparable between the groups. We also considered ongoing extraocular treatments with possible effects on iris motility.

**Conclusions:**

The pupillary size and profile were similar between the groups in mesopic light. Reduced mydriasis was noted in response to light and drugs, while the degree of miosis was reduced in response to inducing drugs in the IC-IOL compared to the control group. This study complements previous results concerning the PC placement of IC-IOLs by adding original observations on drug-induced pupil motility.

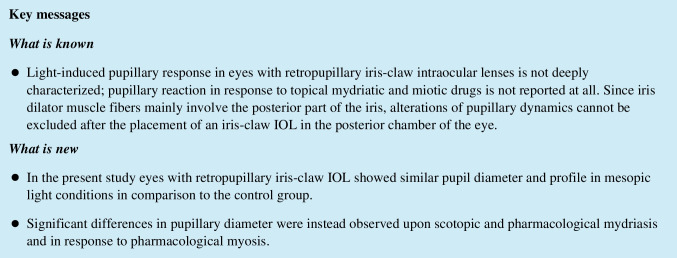

## Introduction

Aphakia can be treated by implantation of an iris-claw intraocular lens (IC-IOL), which allows the crystalline replacement in eyes without the regular capsular support. An IC-IOL may be implanted for the treatment of various conditions, including trauma, complications of cataract surgery, and lens or bag spontaneous luxation in patients with predisposing syndromes (e.g., severe pseudoexfoliation, Marfan syndrome, and Weill-Marchesani syndrome) [1–3]. Although IC-IOLs were originally designed to be clipped in the anterior chamber of the eye, they are increasingly being placed in the posterior chamber (PC) [4, 5]. The possible advantages of PC placement include preserved anatomical compartments of the eye and reduced manipulation of the implant in the anterior chamber with possible lower endothelial damage [6–10]. However, a recent comparative series reported that the decentration of IC-IOLs placed in the anterior chamber was similar to that of regular pseudophakic eyes and lower than that after PC placement [11].

Iris enclavation may lead to some complications of the iris and/or its motility, such as pupil distortion, large iridectomy, and iridodialysis [10]. However, in the vast majority of cases, IC-IOLs are successfully implanted without complications; only a few studies have evaluated light-induced pupillary motility for such cases, and none has evaluated the response of an uncomplicated IC-IOL to pharmacological mydriasis and miosis [12, 13]. The manufacturer’s instructions for an IC-IOL model indicated that anterior implantation of the IC-IOL should involve hooking of “midperipheral virtually immobile iris stroma” by the lens haptics [14]. This statement is not directly applicable for retropupillar enclavation because the iris dilator muscle is mainly located in the posterior part of the iris.

In the present study, we performed high-resolution anterior segment optical coherence tomography (AS-OCT) to measure the pupil size variation in eyes with standard PC implantation of an IC-IOL. The pupillary characteristics and reactions were evaluated under different light conditions and in response to conventional topical mydriatic and miotic drugs. The results were compared with those of the fellow eyes.

## Methods

This cross-sectional study enrolled patients who underwent unilateral implantation of an IC-IOL (Artisan®; Ophtec, Groningen, The Netherlands) at the Ophthalmology unit of the University Hospital of Parma (Parma, Italy). The study protocol was approved by the local ethics committee (#483/2018). The study was conducted in accordance with the Declaration of Helsinki, and written informed consent was obtained from all patients. The study included patients aged ≥ 18 years who underwent PC implantation of an IC-IOL in an aphakic eye 6 months to 3 years before data collection. We excluded patients with history of traumatic aphakia, perioperative IC-IOL disenclavation, postoperative conditions that prevent visualization of the anterior segment, bilateral IC-IOL implantation, extended iris atrophy, or iridodialysis. The medical history of the patients was reviewed to identify drug use that may affect pupillary motility. The patients were examined to record the best corrected visual acuity (BCVA) using the Early Treatment of Diabetic Retinopathy Study chart placed at 4 m, slit-lamp examination findings, intraocular pressure using pneumotonometry, and AS-OCT findings.

### PC placement of the IC-IOL

After removing the remnants of the lens material or unsuitable IOL, if needed, limbal paracentesis was performed at the 3 o’clock position (both study surgeons, PM and ST, are right-handed), and the viscoelastic medium was injected. A clear corneal 5.5-mm incision was made superiorly, and the IC-IOL (with the vault facing down) was inserted into the anterior chamber. The IOL was rotated so that the haptics were located at the 3 and 9 o’clock positions, and then pushed behind the undilated pupil. After making the appropriate centration adjustments, the haptics were alternately slightly pushed against the mid-peripheral iris stroma and enclavated using a smooth microspatula. Finally, the corneal incision was sutured using a non-continuous 10–0 non-absorbable nylon, which was removed 6–9 weeks after surgery. Postoperatively, conventional antibiotics and nonsteroidal anti-inflammatory eye drops were administered for 5 weeks. An Artisan® IOL (Ophtec, Groningen, The Netherlands) was implanted in all cases. The IOL power was selected to achieve emmetropia and was calculated using the SRK/T formula with an A constant of 117.1.

### AS-OCT evaluation

The patients underwent MS-39 imaging (Phoenix v.4.0.1.8; CSO, Florence, Italy) of both eyes. This device performs spectral domain OCT (SD-OCT) and placido-disk corneal topography to provide automated measurements of the anterior segment of the eye. After autocalibration, the scanning tool performs keratoscopy, measurements of the iris front profile for pupil identification, and a series of 25 radial scans within approximately 1 s. The device performs pupillography using a superluminescent light emitting diode source placed at 950 nm and provides an axial resolution of 3.6 μm in tissue and transversal resolution of 35 μm in air [15]. The device is equipped with built-in software for the measurement of the PD under three light conditions: scotopic (S, 0.04 lx), mesopic (M, 4 lx), and photopic (Ph, 50 lx). The quality of the acquired scans was confirmed by the study operators, and measurements were obtained for both eyes. The IC-IOL eyes were included in the IC-IOL group, and the fellow eyes (phakic or pseudophakic) were included in the control group. During mesopic assessment, also pupil circularity was estimated by calculating the ratio between the major and minor axes of the pupil. The PD was then measured with the eyes under pharmacological mydriasis, obtained referring to a known scheme [16]: 1% tropicamide eye drop followed by 10% tropicamide + 0.5% phenylephrine eye drop after 5 and 30 min; the mydriasis assessment was performed after 45 min.

Two weeks later, the examination was repeated with pharmacological miosis of the involved eyes (study and control group) obtained using the instillation of commercial 2% pilocarpine eye drops.

### Statistical analyses

Descriptive statistics (i.e., mean and standard deviation [SD]) are provided for variables of interest. The Shapiro–Wilk test was used to determine the normality of the distribution of the data. A paired *t*-test was used to evaluate differences between the IC-IOL and control groups. Statistical analyses were performed using SPSS (version 28.0.0; IBM Corp, Armonk, NY, USA), and *p* < 0.05 was considered statistically significant.

## Results

In total, 30 patients (18 females and 12 males; mean age: 75 ± 8 years) fulfilled the eligibility criteria and agreed to participate in the study. Each participant had received a retropupillary IC-IOL placement in one eye (16 OD and 14 OS); the fellow eyes were included in the control group (30 eyes: 20 with in-the-bag psudophakia and 10 with a natural lens). The implanted IC-IOLs had an average power of 18.7 ± 1.2 D, and the study visits occurred after a median interval of 18.9 months from surgery. The causes of IC-IOL implantation included a complicated or subluxated cataract (11 eyes, 37%), dislocated/luxated IOL (10 eyes, 33%), luxated nucleus (3 eyes, 10%), IOL opacification (3 eyes, 10%), and long-standing aphakia (3 eyes, 10%). At the time of IC-IOL implantation, a complete parsplana vitrectomy was already present or concomitantly performed in 19 eyes (63%); in the remaining cases, anterior vitrectomy was performed. In total, 12 patients had pseudoexfoliative syndrome (in 3 cases with glaucoma), 2 had open angle glaucoma, and 1 had moderate diabetic retinopathy (all in both eyes).

Table 1 presents the BCVA, mesopic pupil circularity ratio, and PD (mm) under mesopic, photopic, and scotopic light conditions, upon pharmacological mydriasis and miosis in both eyes. The BCVA was higher in the control group compared to the IC-IOL group (*p* = 0.0015). The PD under scotopic conditions and during pharmacological mydriasis was smaller in the IC-IOL group compared to the control group (*p* = 0.0010 and < 0.0001, respectively). During pharmacological miosis, the PD was larger in the IC-IOL group compared to the control group (*p* < 0.0001). The pupil circularity was comparable under basal mesopic conditions.

**Table 1:** Visual acuity and pupillography results (mean ± SD) in the study and control group

	Study eyes	Fellow eyes	*p* value
BCVA (LogMAR)	0.42 ± 0.38	0.17 ± 0.21	0.0015*
SCOTOPIC (0.04 lx) PD (mm)	3.86 ± 0.87	4.43 ± 1.27	0.0010*
MESOPIC (4 lx) PD (mm)	3.52 ± 0.72	3.71 ± 1.12	0.2562
PHOTOPIC (40 lx) PD (mm)	3.18 ± 0.61	3.06 ± 0.93	0.4737
Pharmacological mydriasis PD (mm)	4.51 ± 0.96	5.73 ± 1.35	< 0.0001*
Pharmacological miosis PD (mm)	2.38 ± 0.53	1.8 ± 0.35	< 0.0001*
PD ratio (max/min) Mesopic light	0.94 ± 0.13	0.99 ± 0.05	0.0658

*BCVA* best-corrected visual acuity, *PD* pupil diameter

In total, 8 patients (27%) were taking drugs with miotic potential (oral alpha-lithic, oral amlodipine, oral donepezil, and topical brimonidine in 4, 2, 1, and 1 patient, respectively). Similarly, 8 patients were taking drugs with mydriatic potential (oral selective serotonin reuptake inhibitors, oral pregabalin, oral levodopa, and oral quetiapine in 3, 2, 2, and 1 patients, respectively). One patient received combined therapy with miotic and mydriatic drugs. After excluding this patient, the study parameters did not differ between the IC-IOL eyes from patients treated and not treated with drugs with potential mydriatic or miotic effects.

## Discussion

We investigated the pupillary motility and characteristics in eyes with PC implantation of an IC-IOL and compared them with those of the fellow phakic or pseudophakic eyes. We explored the possible influence of iris enclavation on pupillary shape and motility under basal daylight conditions or mydriasis or miosis in response to light and drug stimulation. We managed the effects of other influencing factors by (a) excluding patients with a history of trauma or extensive iris atrophy or (b) identifying patients with ongoing use of drugs that may affect iris motility.

Under mesopic illumination, the PD and pupillary circularity (ratio between maximal and minimal diameters) were comparable between the groups. The ratio of 0.94 ± 0.13 (ideally set at 1) indicated excellent pupillary circularity in the IC-IOL group. The BCVA was significantly higher in the control group than the IC-IOL group. The comparable PD and pupillary circularity under mesopic conditions between the groups indicated that the possibility that pupil size exceeds the IOL optical zone is very limited. Visible misalignment between the margins of the IC-IOL optic and the pupil was excluded by slit-lamp examination. Therefore, it is likely that the visual acuity of the IC-IOL eyes was negatively affected by the event underlying the aphakia, previous complicated surgery, or the subclinical decentration and tilting of the implant itself [11]. Also, the larger corneal incision required for the iris implant could have played a role in limiting the visual acuity. This is by inducing noticeable against-the-rule astigmatism and/or possible high-order aberrations, both difficult to fully compensate with spectacles. In scotopic light, the PD was smaller in the IC-IOL group than the control group, in line with findings from previous studies [13, 17, 18]. This difference between groups increased after pharmacological mydriasis. Also, pupillary restriction was limited in the IC-IOL group compared to the control group, but this is only after pharmacological stimulation and not under photopic illumination. It has been observed that phacoemulsification itself may induce changes in pupil size regardless of the implanted IOL [19]. This may have introduced bias into our study results since only two-thirds of the control eyes were pseudophakic. However, the presence of the difference between the groups, as well as the increase therein, after pharmacological stimulation suggests that our findings are sustained by iris enclavation. It is possible that the IOL claw pressing the iris tissue mechanically affects the dilator and sphincter muscles, the corresponding nerves, or blood supply, thus limiting the iris motility.

Summarizing, no differences in pupillary size or characteristics were noted in mesopic conditions between the IC-IOL and control groups. By contrast, there was limited mydriasis in response to light and drugs, and limited miosis in response to drugs, in the IC-IOL group compared to the control group. Therefore, the possible undesirable effects of posterior iris hooking may occur in low-light vision or when mydriasis is required for other medical reasons, such as a careful examination of the retinal periphery. The limited miotic response did not affect the adaptation to photopic conditions, thus reducing the risk of symptomatic glare. PC placement of the IC-IOL might counterbalance the reduced pharmacological effect of miotics, if indicated for cases of reduced anterior chamber depth or angular crowding.

Our results related to the pupillary motility and iris profile support the PC placement of IC-IOLs. The results of the present study are in addition to others already available addressing other possible risk profiles of the PC placement of the IC-IOL [9].

## Data Availability

All data and material are available from the corresponding author.
